# *N*-(3-Chloro­phen­yl)-4-methyl­benzamide hemihydrate

**DOI:** 10.1107/S1600536811040992

**Published:** 2011-10-08

**Authors:** Vinola Z. Rodrigues, Lenka Kucková, B. Thimme Gowda, Jozef Kožíšek

**Affiliations:** a Department of Chemistry, Mangalore University, Mangalagangotri 574 199, Mangalore, India; b Institute of Physical Chemistry and Chemical Physics, Slovak University of Technology, Radlinského 9, SK-812 37 Bratislava, Slovak Republic

## Abstract

In the title compound, C_14_H_12_ClNO·0.5H_2_O, the water mol­ecule is located on a twofold axis of symmetry. The *meta*-Cl atom in the aniline ring is positioned *anti* to the N—H bond. The two benzene rings make a dihedral angle of 40.40 (11)°. The crystal structure is stabilized by inter­molecular N—H⋯O and O—H⋯O hydrogen bonds, which link the mol­ecules into chains along the *a* axis.

## Related literature

For the preparation of the title compound, see: Gowda *et al.* (2003 ▶). For studies on the effects of substituents on the structures and other aspects of *N*-(ar­yl)-amides, see: Bowes *et al.* (2003 ▶); Gowda *et al.* (1999 ▶); Rodrigues *et al.* (2011 ▶); Saeed *et al.* (2010 ▶), on *N*-(ar­yl)-methane­sulfonamides, see: Gowda *et al.* (2007 ▶), on *N*-(ar­yl)-aryl­sulfonamides, see: Shetty & Gowda (2005 ▶) and on *N*-chloro-aryl­sulfonamides, see: Gowda & Shetty (2004 ▶). 
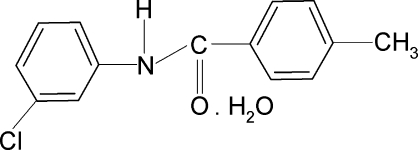


## Experimental

### 

#### Crystal data

C_14_H_12_ClNO·0.5H_2_O
*M* *_r_* = 254.71
Monoclinic, 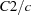

*a* = 7.8078 (3) Å
*b* = 12.1704 (5) Å
*c* = 27.1217 (9) Å
β = 93.564 (3)°
*V* = 2572.24 (17) Å^3^
*Z* = 8
Mo *K*α radiation
μ = 0.29 mm^−1^
*T* = 298 K
0.76 × 0.34 × 0.02 mm

#### Data collection

Oxford Diffraction Xcalibur Ruby Gemini diffractometer
Absorption correction: analytical [*CrysAlis RED* (Oxford Diffraction, 2009 ▶), based on expressions derived from Clark & Reid (1995 ▶)] *T*_min_ = 0.890, *T*_max_ = 0.993
3313 measured reflections
3313 independent reflections
1943 reflections with *I* > 2σ(*I*)
*R*_int_ = 0.040

#### Refinement

*R*[*F* ^2^ > 2σ(*F* ^2^)] = 0.058
*wR*(*F* ^2^) = 0.142
*S* = 1.01
3313 reflections
169 parameters
H atoms treated by a mixture of independent and constrained refinement
Δρ_max_ = 0.31 e Å^−3^
Δρ_min_ = −0.39 e Å^−3^

### 

Data collection: *CrysAlis CCD* (Oxford Diffraction, 2009 ▶); cell refinement: *CrysAlis CCD*; data reduction: *CrysAlis RED* (Oxford Diffraction, 2009 ▶); program(s) used to solve structure: *SHELXS97* (Sheldrick, 2008 ▶); program(s) used to refine structure: *SHELXL97* (Sheldrick, 2008 ▶); molecular graphics: *DIAMOND* (Brandenburg, 2002 ▶); software used to prepare material for publication: *enCIFer* (Allen *et al.*, 2004 ▶).

## Supplementary Material

Crystal structure: contains datablock(s) I, global. DOI: 10.1107/S1600536811040992/bq2309sup1.cif

Structure factors: contains datablock(s) I. DOI: 10.1107/S1600536811040992/bq2309Isup2.hkl

Supplementary material file. DOI: 10.1107/S1600536811040992/bq2309Isup3.cml

Additional supplementary materials:  crystallographic information; 3D view; checkCIF report

## Figures and Tables

**Table 1:** Hydrogen-bond geometry (Å, °)

*D*—H⋯*A*	*D*—H	H⋯*A*	*D*⋯*A*	*D*—H⋯*A*
N1—H1*N*⋯O2^i^	0.86 (2)	2.11 (2)	2.947 (2)	167 (2)
O2—H2*O*⋯O1^ii^	0.84 (2)	1.92 (2)	2.7630 (19)	176 (3)

Symmetry codes: (i) 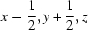; (ii) 
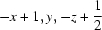
.

**Fig. 1.**
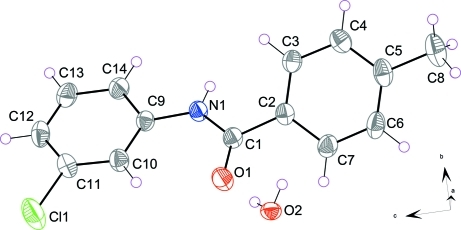
Molecular structure of the title compound showing the atom labeling scheme. Displacement ellipsoids are drawn at the 50% probability level.

**Fig. 2.**
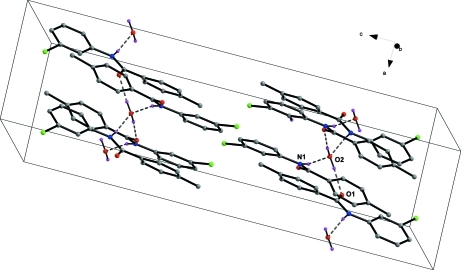
Part of the crystal structure of the title compound. Molecular chains are generated by N—H···O and O—H···O hydrogen bonds which are shown by dashed lines. H atoms not involved in intermolecular bonding have been omitted.

## supplementary crystallographic information

### Comment

The amide and sulfonamide moieties are the constituents of many biologically significant compounds. As part of our studies on the substituent effects on the structures and other aspects of *N*-(aryl)-amides (Bowes *et al.*, 2003; Gowda *et al.*, 1999; Saeed *et al.*, 2010; Rodrigues *et al.*, 2011), *N*-(aryl)-methanesulfonamides (Gowda *et al.*, 2007), *N*-(aryl)-arylsulfonamides (Shetty & Gowda, 2005) and *N*-chloro-arylsulfonamides (Gowda & Shetty, 2004), in the present work, the crystal structure of *N*-(3-Chlorophenyl)- 4-methylbenzamide (I) has been determined (Fig.1).

In (I), the water molecule is in special position and connects the different molecules of the compound. Further, the *meta*-Cl atom in the anilino ring is positioned *anti* to the N–H bond. The N—H and C=O bonds in the C—NH—C(O)—C segment are *anti* to each other, similar to that observed in *N*-(2-chlorophenyl)- 4-methylbenzamide (Rodrigues *et al.*, 2011). The *C*(benzoyl)—NH—C(O)—C(anilino) torsional angle is -172.65 (18)°.

The packing of molecules linked by N1—H1N···O2 and O2—H2O···O1 hydrogen bonds into infinite chains is shown in Fig. 2.

### Experimental

The title compound was prepared according to the method described by Gowda *et al.* (2003). The purity of the compound was checked by determining its melting point. It was characterized by recording its infrared and NMR spectra. Plate like colourless single crystals of the title compound were obtained by slow evaporation of an ethanol solution of the compound (0.5 g in about 30 ml of ethanol) at room temperature.

### Refinement

The C- and N- bound hydrogen atoms were positioned with idealized geometry using a riding model with C–H distances of 0.93Å (C-aromatic), 0.96Å (C-methyl) and N-H = 0.86 Å. The water hydrogen atoms are symmetry related, were seen in difference Fourier maps and were refined as free. The *U*_iso_(H) values were set at 1.2*U*_eq_(C aromatic, N, O) and 1.5*U*_eq_(C methyl). The disordered methyl group was refined using constrain 138

### Crystal data

**Table:**

C_14_H_12_ClNO·0.5H_2_O	*F*(000) = 1064
*M**_r_* = 254.71	*D*_x_ = 1.315 Mg m^−^^3^
Monoclinic, *C*2/*c*	Mo *K*α radiation, λ = 0.71073 Å
Hall symbol: -C 2yc	Cell parameters from 21518 reflections
*a* = 7.8078 (3) Å	θ = 3.5–29.4°
*b* = 12.1704 (5) Å	µ = 0.29 mm^−^^1^
*c* = 27.1217 (9) Å	*T* = 298 K
β = 93.564 (3)°	Plate, colorless
*V* = 2572.24 (17) Å^3^	0.76 × 0.34 × 0.02 mm
*Z* = 8	

### Data collection

**Table:**

Oxford Diffraction Xcalibur Ruby Gemini diffractometer	3313 independent reflections
Radiation source: fine-focus sealed tube	1943 reflections with *I* > 2σ(*I*)
graphite	*R*_int_ = 0.040
Detector resolution: 10.4340 pixels mm^-1^	θ_max_ = 29.5°, θ_min_ = 3.5°
ω scans	*h* = −10→10
Absorption correction: analytical [*CrysAlis RED* (Oxford Diffraction, 2009), based on expressions derived from Clark & Reid (1995)]	*k* = −16→16
*T*_min_ = 0.890, *T*_max_ = 0.993	*l* = −36→33
3313 measured reflections	

### Refinement

**Table:**

Refinement on *F*^2^	Primary atom site location: structure-invariant direct methods
Least-squares matrix: full	Secondary atom site location: difference Fourier map
*R*[*F*^2^ > 2σ(*F*^2^)] = 0.058	Hydrogen site location: inferred from neighbouring sites
*wR*(*F*^2^) = 0.142	H atoms treated by a mixture of independent and constrained refinement
*S* = 1.01	*w* = 1/[σ^2^(*F*_o_^2^) + (0.049*P*)^2^ + 2.1399*P*] where *P* = (*F*_o_^2^ + 2*F*_c_^2^)/3
3313 reflections	(Δ/σ)_max_ < 0.001
169 parameters	Δρ_max_ = 0.31 e Å^−^^3^
0 restraints	Δρ_min_ = −0.39 e Å^−^^3^

### Special details

**Table:**

Experimental. CrysAlis RED (Oxford Diffraction, 2009) Analytical numeric absorption correction using a multifaceted crystal model based on expressions derived (Clark & Reid, 1995).
Geometry. All e.s.d.'s (except the e.s.d. in the dihedral angle between two l.s. planes) are estimated using the full covariance matrix. The cell e.s.d.'s are taken into account individually in the estimation of e.s.d.'s in distances, angles and torsion angles; correlations between e.s.d.'s in cell parameters are only used when they are defined by crystal symmetry. An approximate (isotropic) treatment of cell e.s.d.'s is used for estimating e.s.d.'s involving l.s. planes.
Refinement. Refinement of *F*^2^ against ALL reflections. The weighted *R*-factor *wR* and goodness of fit *S* are based on *F*^2^, conventional *R*-factors *R* are based on *F*, with *F* set to zero for negative *F*^2^. The threshold expression of *F*^2^ > σ(*F*^2^) is used only for calculating *R*-factors(gt) *etc*. and is not relevant to the choice of reflections for refinement. *R*-factors based on *F*^2^ are statistically about twice as large as those based on *F*, and *R*- factors based on ALL data will be even larger.

### Fractional atomic coordinates and isotropic or equivalent isotropic displacement parameters (Å^2^)

**Table:**

	*x*	*y*	*z*	*U*_iso_*/*U*_eq_	Occ. (<1)
C1	0.2326 (2)	0.60328 (17)	0.27192 (7)	0.0465 (5)	
C2	0.2647 (2)	0.63298 (17)	0.21979 (7)	0.0452 (5)	
C3	0.3261 (2)	0.73485 (18)	0.20695 (7)	0.0502 (5)	
H3A	0.3404	0.7896	0.2307	0.060*	
C4	0.3664 (3)	0.7558 (2)	0.15888 (8)	0.0589 (6)	
H4A	0.4091	0.8245	0.1510	0.071*	
C5	0.3444 (3)	0.6770 (2)	0.12247 (8)	0.0606 (6)	
C6	0.2797 (3)	0.5762 (2)	0.13527 (8)	0.0639 (7)	
H6A	0.2615	0.5226	0.1111	0.077*	
C7	0.2413 (3)	0.55318 (19)	0.18318 (8)	0.0566 (6)	
H7A	0.1998	0.4842	0.1910	0.068*	
C8	0.3887 (4)	0.7009 (3)	0.07011 (9)	0.0856 (10)	
H8C	0.423 (2)	0.6369 (10)	0.0554 (3)	0.103*	0.50
H8B	0.476 (2)	0.7518 (13)	0.07042 (9)	0.103*	0.50
H8A	0.2934 (16)	0.7286 (15)	0.0525 (3)	0.103*	0.50
H8F	0.424 (2)	0.6373 (10)	0.0552 (3)	0.103*	0.50
H8E	0.476 (2)	0.7517 (13)	0.07049 (9)	0.103*	0.50
H8D	0.2940 (16)	0.7288 (15)	0.0523 (3)	0.103*	0.50
C9	0.1532 (2)	0.68527 (16)	0.35142 (7)	0.0427 (5)	
C10	0.1959 (3)	0.59716 (19)	0.38225 (7)	0.0528 (5)	
H10A	0.2417	0.5330	0.3699	0.063*	
C11	0.1685 (3)	0.6074 (2)	0.43183 (8)	0.0582 (6)	
C12	0.1025 (3)	0.6999 (2)	0.45181 (8)	0.0623 (6)	
H12A	0.0851	0.7040	0.4854	0.075*	
C13	0.0623 (3)	0.7868 (2)	0.42085 (8)	0.0634 (6)	
H13A	0.0179	0.8509	0.4337	0.076*	
C14	0.0871 (3)	0.78007 (18)	0.37099 (8)	0.0532 (5)	
H14A	0.0592	0.8395	0.3505	0.064*	
N1	0.1767 (2)	0.68533 (15)	0.30024 (6)	0.0446 (4)	
H1N	0.142 (3)	0.7439 (19)	0.2852 (8)	0.051 (6)*	
O1	0.2586 (2)	0.50930 (12)	0.28769 (6)	0.0653 (5)	
O2	0.5000	0.37191 (16)	0.2500	0.0465 (5)	
H2O	0.573 (3)	0.412 (2)	0.2370 (9)	0.077 (8)*	
Cl1	0.22644 (12)	0.49860 (7)	0.47115 (3)	0.0998 (3)	

### Atomic displacement parameters (Å^2^)

**Table:**

	*U*^11^	*U*^22^	*U*^33^	*U*^12^	*U*^13^	*U*^23^
C1	0.0489 (11)	0.0454 (12)	0.0457 (11)	0.0082 (9)	0.0073 (9)	−0.0017 (9)
C2	0.0453 (11)	0.0496 (12)	0.0410 (11)	0.0132 (9)	0.0041 (8)	0.0001 (9)
C3	0.0487 (11)	0.0564 (13)	0.0457 (12)	0.0040 (10)	0.0052 (9)	−0.0042 (10)
C4	0.0569 (13)	0.0665 (15)	0.0544 (14)	0.0037 (11)	0.0113 (10)	0.0077 (11)
C5	0.0581 (13)	0.0809 (18)	0.0433 (12)	0.0259 (12)	0.0080 (10)	0.0040 (12)
C6	0.0819 (16)	0.0681 (17)	0.0413 (12)	0.0271 (13)	0.0003 (11)	−0.0106 (11)
C7	0.0728 (14)	0.0489 (13)	0.0480 (12)	0.0141 (11)	0.0024 (10)	−0.0052 (10)
C8	0.0910 (19)	0.119 (3)	0.0488 (14)	0.0294 (18)	0.0182 (13)	0.0114 (15)
C9	0.0407 (10)	0.0486 (12)	0.0393 (10)	−0.0001 (9)	0.0055 (8)	−0.0030 (9)
C10	0.0592 (13)	0.0557 (13)	0.0443 (12)	0.0075 (10)	0.0083 (9)	−0.0005 (10)
C11	0.0606 (13)	0.0699 (16)	0.0444 (12)	0.0025 (11)	0.0071 (10)	0.0079 (11)
C12	0.0630 (14)	0.0837 (18)	0.0413 (12)	−0.0007 (12)	0.0118 (10)	−0.0086 (12)
C13	0.0734 (15)	0.0627 (15)	0.0552 (14)	0.0057 (12)	0.0145 (11)	−0.0164 (12)
C14	0.0607 (13)	0.0501 (13)	0.0494 (12)	0.0046 (10)	0.0080 (10)	−0.0047 (10)
N1	0.0525 (10)	0.0426 (10)	0.0391 (9)	0.0097 (8)	0.0062 (7)	0.0009 (8)
O1	0.0982 (12)	0.0471 (9)	0.0530 (9)	0.0223 (8)	0.0233 (8)	0.0052 (7)
O2	0.0543 (12)	0.0347 (11)	0.0513 (12)	0.000	0.0100 (10)	0.000
Cl1	0.1414 (7)	0.1010 (6)	0.0589 (4)	0.0332 (5)	0.0211 (4)	0.0276 (4)

### Geometric parameters (Å, °)

**Table:**

C1—O1	1.233 (2)	C8—H8F	0.9223
C1—N1	1.349 (2)	C8—H8E	0.9222
C1—C2	1.495 (3)	C8—H8D	0.9223
C2—C3	1.382 (3)	C9—C14	1.383 (3)
C2—C7	1.393 (3)	C9—C10	1.388 (3)
C3—C4	1.384 (3)	C9—N1	1.411 (2)
C3—H3A	0.9300	C10—C11	1.380 (3)
C4—C5	1.379 (3)	C10—H10A	0.9300
C4—H4A	0.9300	C11—C12	1.365 (3)
C5—C6	1.380 (4)	C11—Cl1	1.742 (2)
C5—C8	1.511 (3)	C12—C13	1.374 (3)
C6—C7	1.380 (3)	C12—H12A	0.9300
C6—H6A	0.9300	C13—C14	1.380 (3)
C7—H7A	0.9300	C13—H13A	0.9300
C8—H8C	0.9223	C14—H14A	0.9300
C8—H8B	0.9223	N1—H1N	0.86 (2)
C8—H8A	0.9223	O2—H2O	0.84 (2)
			
O1—C1—N1	122.76 (19)	C5—C8—H8E	109.4
O1—C1—C2	121.22 (18)	H8C—C8—H8E	109.4
N1—C1—C2	116.01 (18)	H8A—C8—H8E	109.6
C3—C2—C7	118.55 (19)	H8F—C8—H8E	109.1
C3—C2—C1	122.43 (18)	C5—C8—H8D	109.9
C7—C2—C1	118.9 (2)	H8C—C8—H8D	109.4
C2—C3—C4	120.4 (2)	H8B—C8—H8D	109.1
C2—C3—H3A	119.8	H8F—C8—H8D	109.2
C4—C3—H3A	119.8	H8E—C8—H8D	109.2
C5—C4—C3	121.4 (2)	C14—C9—C10	119.64 (18)
C5—C4—H4A	119.3	C14—C9—N1	116.84 (18)
C3—C4—H4A	119.3	C10—C9—N1	123.51 (18)
C4—C5—C6	118.0 (2)	C11—C10—C9	118.1 (2)
C4—C5—C8	120.9 (3)	C11—C10—H10A	120.9
C6—C5—C8	121.1 (2)	C9—C10—H10A	120.9
C5—C6—C7	121.4 (2)	C12—C11—C10	123.1 (2)
C5—C6—H6A	119.3	C12—C11—Cl1	118.31 (17)
C7—C6—H6A	119.3	C10—C11—Cl1	118.56 (19)
C6—C7—C2	120.2 (2)	C11—C12—C13	118.0 (2)
C6—C7—H7A	119.9	C11—C12—H12A	121.0
C2—C7—H7A	119.9	C13—C12—H12A	121.0
C5—C8—H8C	109.5	C12—C13—C14	120.8 (2)
C5—C8—H8B	109.5	C12—C13—H13A	119.6
H8C—C8—H8B	109.5	C14—C13—H13A	119.6
C5—C8—H8A	109.5	C13—C14—C9	120.2 (2)
H8C—C8—H8A	109.5	C13—C14—H14A	119.9
H8B—C8—H8A	109.5	C9—C14—H14A	119.9
C5—C8—H8F	110.0	C1—N1—C9	128.76 (18)
H8B—C8—H8F	109.2	C1—N1—H1N	116.7 (14)
H8A—C8—H8F	109.3	C9—N1—H1N	114.3 (14)
			
O1—C1—C2—C3	−144.7 (2)	C14—C9—C10—C11	0.7 (3)
N1—C1—C2—C3	34.3 (3)	N1—C9—C10—C11	179.3 (2)
O1—C1—C2—C7	31.5 (3)	C9—C10—C11—C12	−0.3 (3)
N1—C1—C2—C7	−149.48 (19)	C9—C10—C11—Cl1	−178.47 (16)
C7—C2—C3—C4	−1.2 (3)	C10—C11—C12—C13	−0.3 (4)
C1—C2—C3—C4	174.99 (18)	Cl1—C11—C12—C13	177.90 (18)
C2—C3—C4—C5	0.9 (3)	C11—C12—C13—C14	0.5 (4)
C3—C4—C5—C6	0.4 (3)	C12—C13—C14—C9	−0.1 (3)
C3—C4—C5—C8	179.9 (2)	C10—C9—C14—C13	−0.5 (3)
C4—C5—C6—C7	−1.5 (3)	N1—C9—C14—C13	−179.24 (19)
C8—C5—C6—C7	179.0 (2)	O1—C1—N1—C9	6.4 (3)
C5—C6—C7—C2	1.2 (3)	C2—C1—N1—C9	−172.65 (18)
C3—C2—C7—C6	0.2 (3)	C14—C9—N1—C1	−177.4 (2)
C1—C2—C7—C6	−176.16 (19)	C10—C9—N1—C1	3.9 (3)

### Hydrogen-bond geometry (Å, °)

**Table:**

*D*—H···*A*	*D*—H	H···*A*	*D*···*A*	*D*—H···*A*
N1—H1N···O2^i^	0.86 (2)	2.11 (2)	2.947 (2)	167 (2)
O2—H2O···O1^ii^	0.84 (2)	1.92 (2)	2.7630 (19)	176 (3)

Symmetry codes: (i) *x*−1/2, *y*+1/2, *z*; (ii) −*x*+1, *y*, −*z*+1/2.
